# Genetic Diversity and Demographic History of *Cajanus spp.* Illustrated from Genome-Wide SNPs

**DOI:** 10.1371/journal.pone.0088568

**Published:** 2014-02-12

**Authors:** Rachit K. Saxena, Eric von Wettberg, Hari D. Upadhyaya, Vanessa Sanchez, Serah Songok, Kulbhushan Saxena, Paul Kimurto, Rajeev K. Varshney

**Affiliations:** 1 International Crops Research Institute for the Semi-Arid Tropics (ICRISAT), Hyderabad, Andhra Pradesh, India; 2 Department of Biological Sciences, Florida International University, Miami, Florida, United States of America; 3 Fairchild Tropical Botanic Garden, Kushlan Institute for Tropical Science, Miami, Florida, United States of America; 4 Florida International University, Department of Earth and Environment, Miami, Florida, United States of America; 5 Egerton University, Egerton, Kenya; National Institute of Plant Genome Research, India

## Abstract

Understanding genetic structure of *Cajanus* spp. is essential for achieving genetic improvement by quantitative trait loci (QTL) mapping or association studies and use of selected markers through genomic assisted breeding and genomic selection. After developing a comprehensive set of 1,616 single nucleotide polymorphism (SNPs) and their conversion into cost effective KASPar assays for pigeonpea (*Cajanus cajan*), we studied levels of genetic variability both within and between diverse set of *Cajanus* lines including 56 breeding lines, 21 landraces and 107 accessions from 18 wild species. These results revealed a high frequency of polymorphic SNPs and relatively high level of cross-species transferability. Indeed, 75.8% of successful SNP assays revealed polymorphism, and more than 95% of these assays could be successfully transferred to related wild species. To show regional patterns of variation, we used STRUCTURE and Analysis of Molecular Variance (AMOVA) to partition variance among hierarchical sets of landraces and wild species at either the continental scale or within India. STRUCTURE separated most of the domesticated germplasm from wild ecotypes, and separates Australian and Asian wild species as has been found previously. Among Indian regions and states within regions, we found 36% of the variation between regions, and 64% within landraces or wilds within states. The highest level of polymorphism in wild relatives and landraces was found in Madhya Pradesh and Andhra Pradesh provinces of India representing the centre of origin and domestication of pigeonpea respectively.

## Introduction

Understanding the germplasm diversity and relationships among breeding material is critical to crop improvement. Wild relatives of crops are crucial reservoirs of natural diversity, often possessing abiotic stress tolerance, disease resistance, and other characters that are absent or inadequate in breeding material. Natural selection, domestication and centuries long breeding practices for desirable traits have resulted in a loss of genetic diversity in most annual crop species [1]–[5] and this seems to be more severe in self-pollinated or partially out crossing species such as chickpea (*Cicer arietinum*) [6] and pigeonpea (*Cajanus cajan*) [7]–[9]. Wild relatives and landraces are the best source for increasing diversity in the breeding material as they can be crossed, albeit sometimes with some difficulty, into cultivated forms [2], [10]. There are secondary and tertiary gene pools which can contribute to crop improvement, but may consist of several closely related species-complexes [11], [12] and may require extensive work to cross into the cultivated gene pool. In many cases we know very little about the ecology and population biology of these taxa in their natural habitats, and species delineation may be rudimentary for most crop wild relatives. Characterization of these resources is critical, as it can identify regions of diversity, and suggest areas where greater collections would be helpful.

Levels of genetic variation present in different wild relatives of a crop may vary due to different distributions and evolutionary histories. In species complexes related to crops, some clades may have colonized new areas relatively recently, such as since the last glaciation, and may have undergone colonization bottlenecks in that process [9], [13], [14]. These processes are poorly understood in most crop wild relatives, but may have a significant impact on the value of wild relatives for breeding programs. We can improve our understanding of the relationship of wild species to cultivated forms by localizing the region of domestication, even in cases where the wild progenitor is clear. If the wild progenitor varies spatially, the crop may most closely resemble the wild populations from a particular region, and may show evidence of multiple regions of domestication [15]. However, the signal of regional contribution to domesticated material depends on the scale of sampling and the pace and intensity of domestication [16], [17]. Spatial variation in wild relatives also may serve as a bridge for introgression, allowing more distant relatives to be crossed into an intermediate that is compatible with the cultivated form. Finally, variation in wild relatives may also give us insight into locally adaptive variants in wild species that can be harnessed to provide local adaptation to a crop [18]. Archaeological evidence, high diversity of wild species and cultural usage have supported India as the domestication centre of pigeonpea [19], [20]. This evidence is further supported by recent molecular studies that are providing insights in to pigeonpea domestication [9].

Cultivated pigeonpea suffers from low levels of genetic diversity [21] and existing genetic diversity in wild relatives has received relative little attention or limited systematic use [22]. In order to broaden the genetic diversity in the cultivated gene pool, it is imperative to understand the genetic diversity present in wild relatives in a systematic manner with the genome wide markers. In the past a number of marker systems such as random amplified polymorphic DNA (RAPD) [23], diversity array technology markers (DArT) [24] and simple sequence repeats (SSRs) [21] have been used for detecting genetic diversity in the cultivated gene pool and limited number of wild relatives. Single nucleotide polymorphisms (SNPs) are now markers of choice for various genome wide analysis due to their higher levels of polymorphism, accuracy and automated genotyping methods [25]. A number of high-throughput SNP genotyping platforms are available for the community to make SNP genotyping cost-effective such as BeadXpress and GoldenGate assays from Illumina Inc. Many of these platforms have been developed and used in several crop species like barley [26], wheat [27], maize [28] oilseed rape [29], soybean [30], cowpea [31] and pea [32]. Such platforms, however, not found cost-effective when a variable number of SNPs are required for a number of applications in the same species with a variable size of genotypes. In such cases, **C**ompetitive **A**llele **S**pecific **P**CR (KASPar) assay from KBiosciences (www.kbioscience.co.uk) seems to be an effective marker assay. Because of the importance of KASPar assays in SNP genotyping more samples with a few SNPs, they have been developed in wheat [33], common bean [34], chickpea [35], pigeonpea [8] and recently in peanut [36].

**Figure 1:**
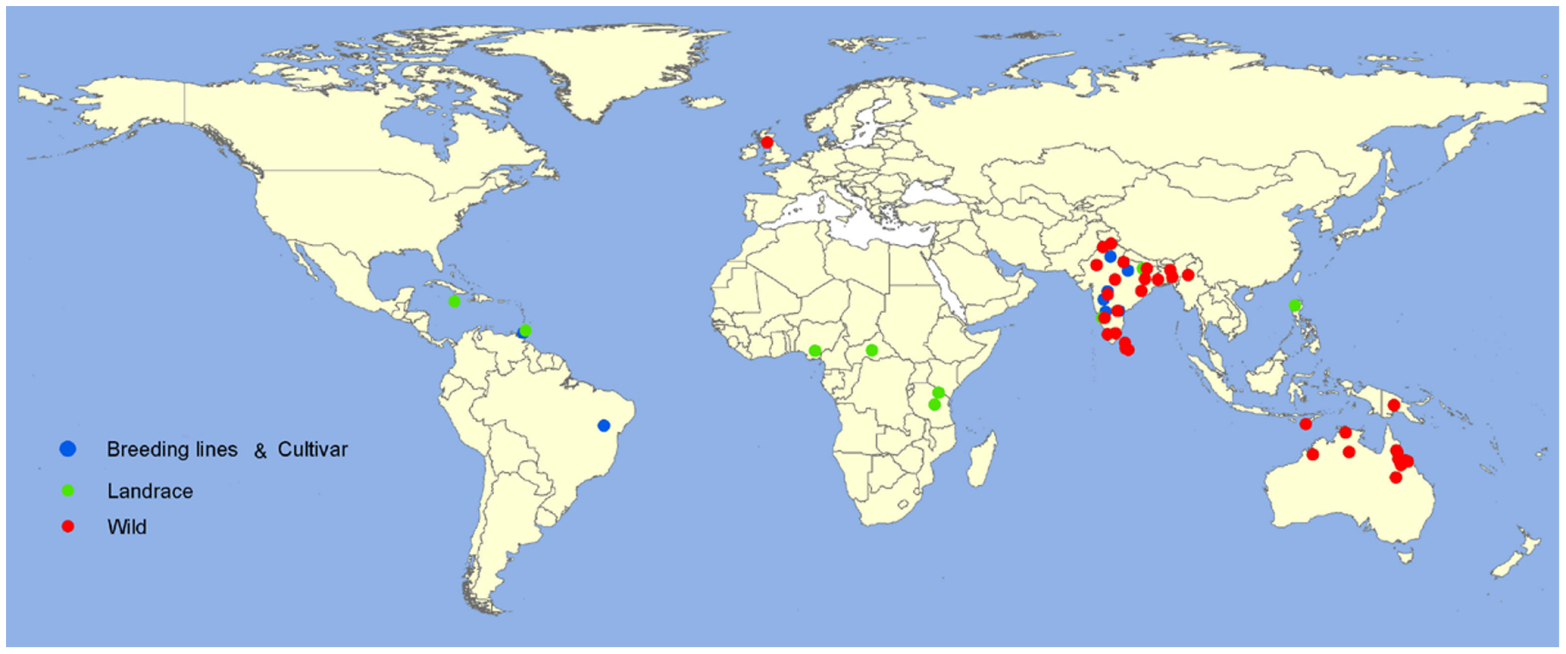
Geographical distribution of the collection sites for cultivated and wild *Cajanus* accessions.

This study reports the genetic diversity and insights in to *Cajanaus* origin using a broad panel of 184 genotypes representing 18 *Cajanus* species across the primary (77), secondary (69) and tertiary gene pools (38), as well as cultivated germplasm from three continents (Figure 1) representing a range of forms from landraces to elite breeding materials using 1,616 SNP markers through KASPar genotyping platform.

## Methods

### Germplasm and DNA isolation

A total of 184 accessions representing 18 *Cajanus* species were selected from >13,000 *Cajanus* accessions deposited in GeneBank and parental lines of mapping populations (Table S1). Total DNA was isolated from two to three young leaves following a standard DNA isolation protocol [37]. The DNA quantity for each sample was assessed on 0.8% agarose gel.

### Single nucleotide polymorphism and KASPar genotyping

SNPs were identified by using next generation sequencing (Illumina GA IIx) technology on 12 parental genotypes of mapping populations [8]. In brief a total of 128.9 million, 36 bp short single end reads were generated from these genotypes. Subsequently SNPs were identified by aligning of sequence reads generated from each of the counter genotypes against the reference assembly, i.e pigeonpea transcriptome assembly that was developed by Kudapa et al. [38]. High quality SNPs were selected for **C**ompetitive **A**llele **S**pecific **P**CR (KASPar) assay from KBiosciences assay and pigeonpea specific assays were developed as described in Saxena et al. [8].

### Data analysis

To assess genetic diversity within groups formed on the basis of biological status (passport data) and geographical origin, we used Genalex 6.3 [39] to estimate observed heterozygosity (Ho), expected heterozygosity (He), fixation index (Fst), and % polymorphism. We subdivided the germplasm several ways: as primary, secondary and tertiary gene pools; as wild species, landraces, and breeding lines, and geographically by continent, country, and within India, by region and state. Based on these categories, we hierarchically analyzed variation with an Analysis of Molecular Variance (AMOVA), implemented in Genalex 6.3. We assessed spatial variation in the groups of germplasm by calculating spatial autocorrelation, implemented in Genalex 6.3. In a complementary analysis, SNPs having mapping positions were used to assess gene diversity according to l1 linkage groups [8] in wild species, landraces, breeding lines and across the germplasm by using PowerMarker software (http://statgen.ncsu.edu/powermarker/). The polymorphism information content or PIC values for developed makers across 184 accessions were calculated by using PowerMarker software (http://statgen.ncsu.edu/powermarker/).

As our analysis of the germplasm depends on the accuracy of the passport data, we verified the groupings by STRUCTURE analyses [40]. We did this with the primary, secondary, and tertiary gene pools, and with the Indian landraces and wilds in two separate STRUCTURE analyses. For both sets of analyses, we ran STRUCTURE on our full dataset of 1,616 SNPs without mapping information, using an admixture model and the default settings. We used Structure harvester [41] and the Evanno method [42] to determine the most likely number of populations (k) present in a sample. To cluster the genetic variation, we also performed a principal component analysis in Genalex 6.3 [39]. Pairwise relatedness was calculated as genetic distance with Genalex 6.3 [39]. The matrix of genetic distances was used to create a neighbour-joining tree with Mega 5.05[43].

## Results

### SNP marker polymorphism

**Figure 2:**
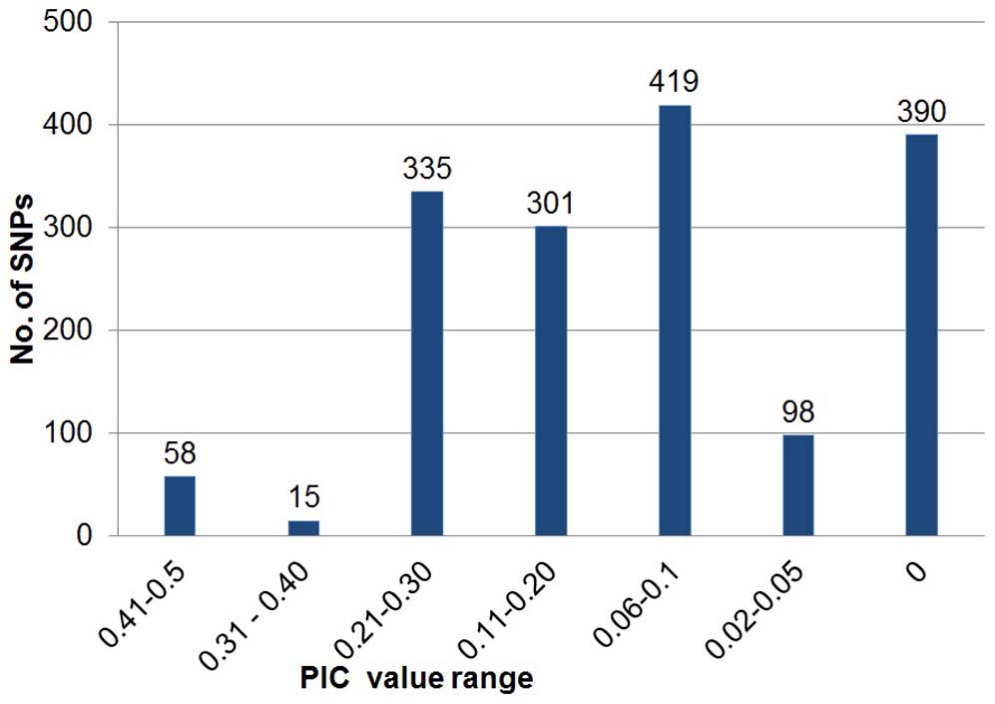
Polymorphism information content (PIC) value range of 1,616 PKAM screened over 184 *Cajanus* accessions.

A total of 1,616 SNPs were used for polymorphism screening on 184 *Cajanus* accessions representing cultivated *C. cajan* (77 accessions) and its wild relatives (107 accessions) (Table S1). The wild accessions represent 18 wild relative species taxonomically placed in gene pool II (GP II) and gene pool III (GP III). The cultivated accessions include elite cultivars and landraces. All the sampled accessions in this study representing widespread geographical regions, ranging from Africa, Asia, Latin America and Australia (Figure 1). From entire set of SNPs we used, 1,615 and 1,504 could be amplified in GP II and GP III respectively (Table 1). A total of 1,226 markers from the set of 1,616 markers were found to be polymorphic across 184 *Cajanus* accessions (Table S2). The polymorphic information content (PIC) for the 1,226 markers ranged from 0.02 to 0.50, with an average of 0.16 for all examined accessions (Figure 2). In the case of cultivated accessions 210 markers were found polymorphic, whereas 1,016 SNPs were polymorphic among the wild accessions.

**Table 1:** SNP marker polymorphism status across cultivated and wild *Cajanus* accessions.

	Cultivated (77)		Wild (107)	
	Breeding lines (56)	Landraces (21)	Gene pool II (69)	Gene pool III (38)
No. of markers used	1616	1616	1616	1616
No. of markers amplified	1616	1616	1615	1504
No. of polymorphic markers	134	210	1181	722
Average PIC value of polymorphic markers	0.19	0.17	0.24	0.24
Average gene diversity of polymorphic markers	0.24	0.2	0.29	0.3
Average diversity across	0.01	0.02	0.26	0.2

### Genetic diversity in *Cajanus*

SNP genotyping data obtained for all polymorphic markers on 184 accessions were used to assess the genetic diversity harboured within the germplasm. The average gene diversity across the 56 breeding lines was lowest (0.01) followed by 21 landraces (0.02). In wild relatives, 69 accessions from GP II have a higher (0.26) gene diversity as compare to 38 accessions from GP III (0.2) (Table 1). By using the SNP genotyping data, gene diversity, as measured by expected heterozygosity (He), ranged from 0.022 in GP I to 0.214 in GP II (Table 2). In the case of breeding lines, landraces and wild relatives expected heterozygosity (He) was estimated as 0.02, 0.027 and 0.2 respectively (Table S3). To estimate the gene diversity at the level of linkage groups (LGs) across the breeding lines, landraces and wild relatives, 875 mapped markers were used. Across 184 accessions the average gene diversity of these mapped markers was 0.35, whereas it was highest in wild relatives (0.26) followed by landraces (0.02) and breeding lines (0.01) (Table 3). While comparing average gene diversity of the mapped markers on the individual LGs, all the LGs showed loss of gene diversity during the course of domestication (wild relatives to landraces) and selection (landraces to breeding lines) (Figure S1). Interestingly, average gene diversity in CcLG06 was the most differentiated among the wild relatives (0.263), landraces (0.003) and breeding lines (0.00) (Table 3).

**Table 2:** Diversity in three different gene pools (GP) of pigeonpea germplasm.

GP	Sample size		N	Na	Ne	I	Ho	He	UHe	F	%P
GP I	77	Mean	76.277	1.154	1.037	0.036	0.01	0.022	0.022	0.679	15.41%
		SE	0.042	0.009	0.004	0.003	0.002	0.002	0.002	0.013	
											
GP II	69	Mean	43.39	1.730	1.342	0.333	0.013	0.214	0.217	0.928	73.08%
		SE	0.639	0.011	0.008	0.006	0.001	0.004	0.004	0.005	
											
GP III	38	Mean	22	1.377	1.146	0.206	0.006	0.133	0.136	0.935	44.68%
		SE	0.396	0.015	0.011	0.006	0.001	0.004	0.004	0.005	

Na  =  No. of Different Alleles, Ne  =  No. of Effective Alleles  =  1 / (Sum pî2), I  =  Shannon's Information Index  =  −1* Sum (pi * Ln (pi)), Ho  =  Observed Heterozygosity  =  No. of Hets / N, He  =  Expected Heterozygosity  =  1 - Sum pî2, UHe  =  Unbiased Expected Heterozygosity  =  (2N / (2N-1)) * He, F  =  Fixation Index  =  (He − Ho) / He  =  1 − (Ho / He) (Where pi is the frequency of the ith allele for the population & Sum pî2 is the sum of the squared population allele frequencies), %P =  percent of loci polymorphic.

**Table 3:** Gene diversity across breeding lines, landraces and wild relatives estimated by the 875 mapped PKAM.

Linkage group	Gene diversity			
	Breeding lines	Landraces	Wild	Across
CcLG01	0.007	0.012	0.253	0.320
CcLG02	0.007	0.019	0.357	0.357
CcLG03	0.006	0.020	0.243	0.351
CcLG04	0.019	0.027	0.252	0.359
CcLG05	0.009	0.021	0.253	0.299
CcLG06	0.000	0.003	0.263	0.368
CcLG07	0.022	0.032	0.243	0.370
CcLG08	0.009	0.018	0.243	0.350
CcLG09	0.017	0.034	0.291	0.388
CcLG10	0.017	0.028	0.277	0.347
CcLG11	0.021	0.029	0.271	0.376
Average	0.012	0.022	0.268	0.353

### Relatedness of cultivated and wild species

**Figure 3:**
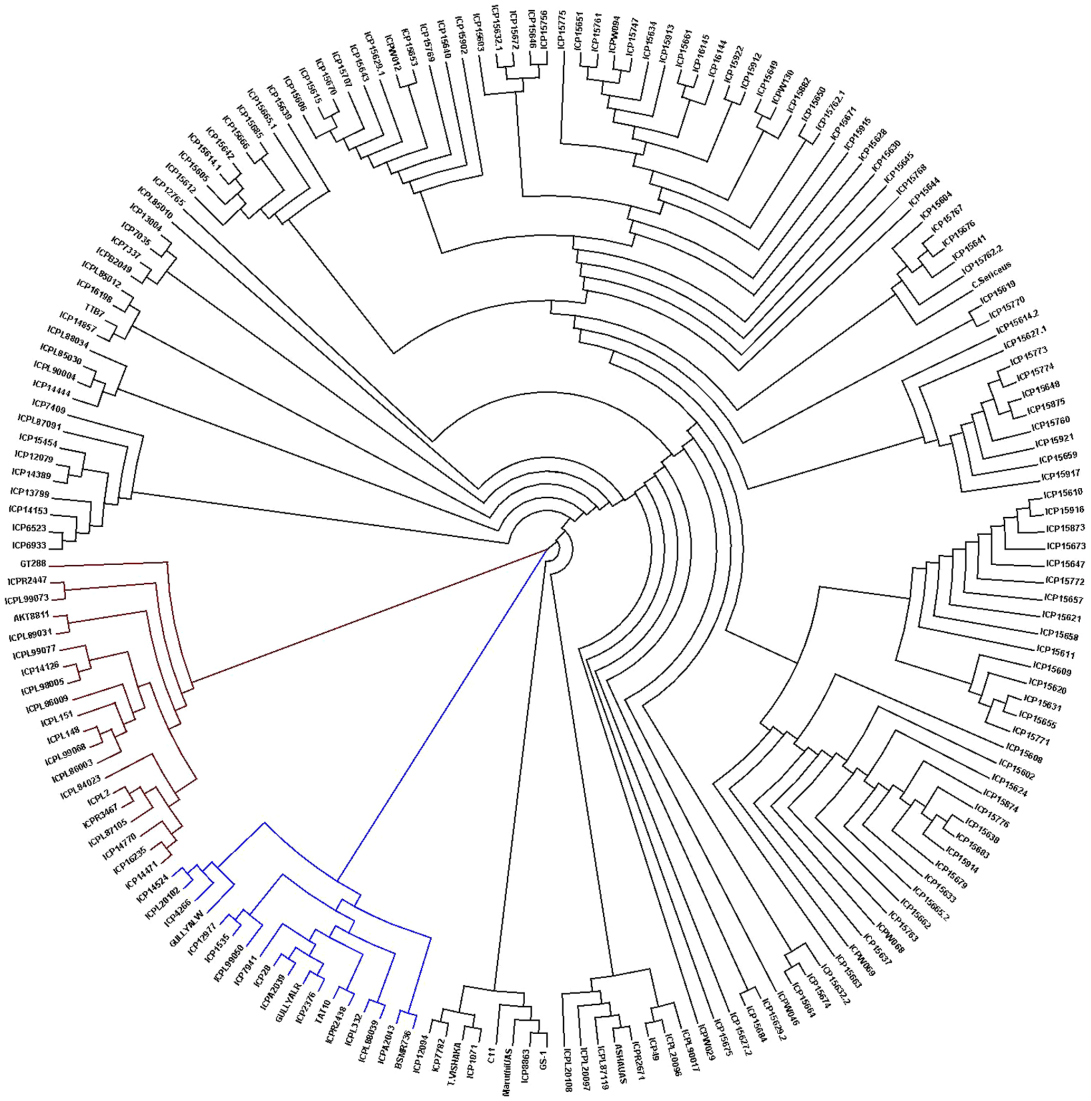
Neighbor-joinging tree of pairwise relatedness among 184 accessions.

Because breeders often use a limited range of material, assessing the relatedness of cultivars in germplasm collections can assist with selecting distantly related lines for breeding programs. For this purpose, we present pairwise relatedness through neighbour-joining trees based on pairwise genetic distances (Figure 3). All 184 accessions were classified into three main clusters: cluster ‘I’, cluster ‘II’ and cluster ‘III’. Cluster ‘I’ contained 18 cultivated accessions; cluster ‘II’ contained 20 cultivated accessions while the remaining 146 cultivated and wild accessions were grouped in cluster ‘III’. Under each of the main clusters, accessions were grouped further into sub-clusters. It is interesting to note that cluster ‘I’ and cluster ‘II’ were made up solely of cultivated accessions, whereas, in cluster ‘III’ cultivated accessions were grouped together with the wild relatives. For instance, 13 breeding lines and 5 landraces were grouped in cluster ‘I’ and 18 breeding lines and 2 landraces were grouped in cluster ‘II’. In the case of cluster ‘III’ 107 wild accessions representing 18 wild relative species were grouped together with the 25 breeding lines and 14 landraces. Accessions from 18 wild relative species were found scattered and no clear grouping could be detected in cluster ‘III’. In order to check the effect of possible cross pollination on varietal maintenance, SNP genotyping data was also used to detect the heterogeneity present in two leading varieties (ICPL 87119 or ASHA and ICP 8863 or Maruthi). It was anticipated that there could be variation from plant to plant at the genome level, and hence samples were collected from two different sources (ICRISAT-Patancheru and UAS-Bangalore). No significant differences were identified and both samples from this variety grouped in close proximity in cluster ‘III’.

We used STRUCTURE to assess the clustering of cultivated and wild genotypes. STRUCTURE divided the wild and cultivated accessions into two groups, representing cultivated and wild gene pools. Several wild lines did show evidence of admixture with cultivated material. To further assess relationships among accessions we separated the accessions into gene pools. When the germplasm in the primary, secondary, and tertiary genepools was analyzed with STRUCTURE, the three gene pools were classified into just two groups, with the primary gene pool distinguished from the secondary and tertiary gene pools (Figure S2a). We also conducted a Principal Coordinate Analysis (PCoA) to distinguish among the primary, secondary, and tertiary gene pools. Accessions representing GP I clustered in a tight group, whereas accessions from GP II and GP III were scattered about. We found substantial overlap among the gene pools. The first two discriminant axes accounted for 76% and 10% of the genetic variation, respectively (Figure S2b).

### Regional patterns of variation

In order to find the regional patterns of variation, landraces and wild accessions were classified by their continent, country and province of origin. At the continental scale, accessions were grouped as Meso America, South Asia, sub-Saharan Africa and Australia-Oceania. The highest per cent polymorphism was identified within landraces (79.76%) and wild relatives (96.60%) present in South Asia. Variation measured by expected heterozygosity (0.48 in wilds and 0.38 in landraces) was highest in South Asia (Table S4). Analysis of Molecular Variance (AMOVA) was used to partition variance among hierarchical sets of landraces and wild species. At the continental scale 69% of the variation segregated between landraces and wilds, and 31% within continents, with no variation among continents (Figure S3).

**Figure 4:**
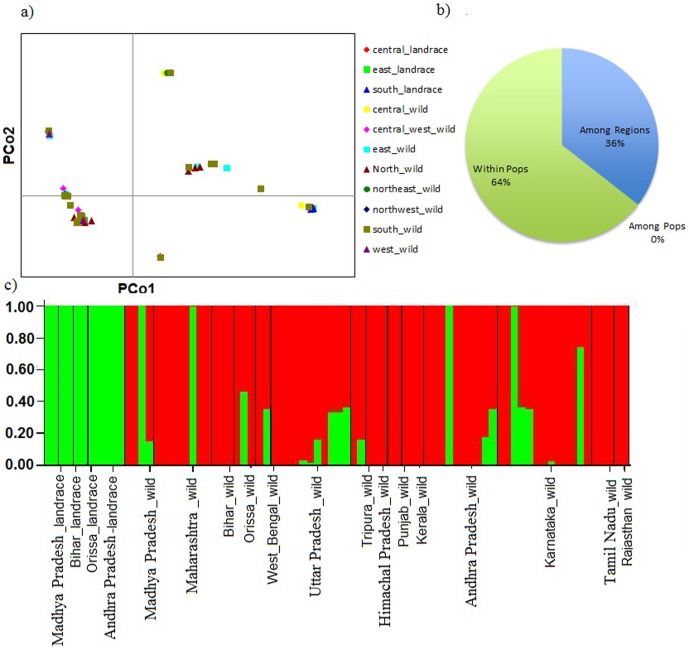
Population analysis of *Cajanus* accessions present in Indian regions and provinces *a)* Principal coordinates analysis of domesticated pigeonpea and wild relatives in 11 defined zones *b)* Analysis of molecular variance (AMOVA) in 11 defined zones *c)* Structure results across gene pools at the province scale

To further asses the regional diversity at the country scale, accessions were grouped as India, Tanzania, Myanmar, Sri Lanka, Australia and Papua New Guinea. The highest level of polymorphism was observed within wild relatives (96.47%) and landraces (76.49%) present in India (Table S5). Similarly expected heterozygosity was found to be highest in wild relatives (0.48) and landraces (0.38) originating in India. These results verify the previous postulations of India being the centre of origin and primary domestication centre [9], [19], [20]. Genetic polymorphism was highest in wild and landrace groups of Indian origin, although surprising amounts of landrace variation were present in some of the landrace material from Meso America and sub-Saharan Africa as well. Further attempts were made to narrow down and mark the centre of origin and domestication within India; accessions from India were grouped according to province (Table S6). Genetic polymorphism within wild relatives were found to be highest in Andhra Pradesh (93.50%) followed by Madhya Pradesh (92.45%) as compare to other provinces in India. We also found the highest polymorphism in Andhra Pradesh (75.43%) followed by Madhya Pradesh (75.31%). The remainder of the South Indian landraces had greater diversity than landraces from other regions of India. Among Indian regions and province within regions, we found 36% of the variation between regions, and 64% within landraces or wilds within provinces, with no variation among provinces (Figure 4). A further principal coordinate analysis of the Indian landrace and wild material did not cluster genotypes by region or wild/landrace (Figure 4). To investigate genetic relationships among accessions and to search for evidence of genetic admixture between landraces and wild accessions, we performed a further STRUCTURE analysis on material from different provinces of India. At a K of 2, the wild species and landraces from different provinces consistently shared partial genetic composition (Figure 4). Landraces from Madhya Pradesh, Bihar, Orissa and Andhra Pradesh clearly separated from their wild ancestors. The genetic composition of wild relatives from different provinces had shown admixture in few accessions which were potentially the progenitor of these landraces. This shared genetic composition is not unexpected as domesticated *C. cajan* is derived from the wild accessions from India.

Several studies have shown that the highest heterozygosity is present in accessions from centre of origin [44]. The maximum expected heterozygosity found in wild relatives was 0.49 within the accessions from Madhya Pradesh and 0.47 in Andhra Pradesh (Table S6). It is important to mention here that size of the analysed samples was highly variable and low. As Madhya Pradesh was represented by only two accessions from landraces and two wild relative species (three accessions from *C. cajanifolius* and one accession from *C. scarabaeoides*) and Andhra Pradesh had five accessions from landraces and 10 accessions from wild relatives representing five species (*C.albicans, C.cajanifolius, C.crassus, C.scarabaeoides* and *C.sericeus*). However, based on current sampling, the higher heterozygosity is consistent with Madhya Pradesh being the centre of origin of pigeonpea. Expected heterozygosity in landraces was similar (0.37) in both the states (Table S6). Here it might be a function of sampling size used for the current study.

## Discussion

This study reports the patterns of variation in cultivated pigeonpea and its wild relatives using SNP markers. Polymorphism survey of sampled *Cajanus* accessions indicated that cultivated pigeonpea is missing significant genetic diversity that was found in wild relatives. The wild relatives of pigeonpea remain the most critical source for increasing the available variation for pigeonpea breeding [45], even if their use has been limited due to a combination of poor agronomic traits, incomplete characterization, and limited collections.

### Utility of KASPar assays for germplasm charterization

A number of marker systems have been developed for pigeonpea such as random amplified polymorphic DNA (RAPD) [23], amplified fragment length polymorphism (AFLP) [46], diversity array technology markers (DArT) [47], single feature polymorphism (SFP) [48] and simple sequence repeats (SSRs) [21]. Recently SNPs markers have also been developed and converted to cost effective genotyping platforms such as KASPar (PKAM [8]: **P**igeonpea **K**aspar **A**ssay **M**arkers) and BeadXpress assays [49]. KASPar assays provide flexibility in terms of number of SNPs used for genotyping. This feature provides upper edge to KASPar assays as compared to other SNP genotyping assays such as BeadXpress and Infinium assays. KASPar assays have been used for linkage mapping and parental polymorphism estimation [8], however these assays have not been used for large scale germplasm characterization in pigeonpea. KASPar assays have been found suitable for diversity estimation in common bean [34], chickpea [35] and peanut [36]. In the present study 75.86% PKAMs were found polymorphic while screening on 184 *Cajanus* accessions representing elite breeding lines, landraces and wild relatives, which is fractionally short from parental polymorphism identified in 24 pigeonpea genotypes (77.4%) [8] and peanut (80%) [36] and higher than chickpea (66.8%) [35]. PKAM categorization of germplasm agrees with the previous analysis of extent of diversity present in cultivated pool and wild relatives of pigeonpea conducted with AFLP [46] and DArT [24] markers. In terms of sub-divisions of *Cajanus* accessions, PKAM allowed the identification of two separate clusters corresponding to cultivated pigeonpea and one cluster corresponding to both wild relatives and cultivated pigeonpea. No clear groupings were identified in terms of genepools, however in cluster ‘III’, GP I accessions showed sub-grouping. GP II and GP III accessions were scattered in the cluster ‘III’. Nevertheless, the *Cajanifolius* wild genotypes were closer to the cultivated pigeonpea than other wild species as revealed in previous marker based studies [9], [50].

### Variation across linkage groups

Great strides have been made in both sequencing the pigeonpea genome [51] and in placing a range of markers from SSRs to ESTs onto the linkage groups [21], [38], [47]. This study has assisted in the next step in providing information on sampled loci across the pigeonpea genome harboring high diversity. These sites may harbor unique features, from loci under different forms of natural selection to locations of inversions as discovered in case of chickpea by re-sequencing of cultivated and wild accessions [6]. Genotyping data suggested major loss of diversity across the pigeonpea genome during the course of domestication and further by modern breeding. These findings indicate that the cultivated pigeonpea has a narrowed genetic reservoir and possibly a reduced capacity to respond to future needs. Therefore, new methods must be applied to reintroduce adaptive diversity lost through domestication and breeding. This study emphasizes the need for support and planning for on-going, new, or novel efforts to maintain genetic diversity using wild relatives. Future crop production challenges will include new or more virulent diseases, environmental changes, degradation of agricultural land, etc., necessitating alternatives. Therefore, a diverse genetic reservoir in crop production remains as crucial as ever.

### Insights into domestication

This study used high-throughput SNP genotyping for investigating the genetic diversity in cultivated pigeonpea and its wild relatives towards understanding the domestication and centre of origin. These analysis have provided better understanding about the genetic diversity present in *Cajanus* as compared to previous studies [8], [9], [24], [49]. This study was in congruence with some of the previous findings based on Archaeological [19], [20] and molecular evidence [9] supported India as the domestication centre of pigeonpea. These results also assigned *C. cajanifolius* as the closest wild relative of cultivated pigeonpea and most likely progenitor species. Based on genetic diversity and heterozygosity, in the present study Madhya Pradesh (central province in India) has been designated as centre of origin of pigeonpea, however, almost similar levels of diversity were found in both wild relatives and landraces in the two Indian states namely Andhra Pradesh and Madhya Pradesh. Andhra Pradesh and Madhya Pradesh have been designated as centre of domestication and centre of origin respectively in past [19], [20]. However, our sample sizes were restricted by the size of existing collections of wild relatives and primitive landraces, and were insufficient to have complete confidence in Andhra Pradesh being the centre of domestication or diversification. Even if Andhra Pradesh or a nearby state is the centre of domestication, likely other regions, such as the more topologically and edaphically diverse Western Ghats region of India were also important areas of diversification of wild *Cajanus* species. And the relatively open breeding system of cultivated *C. cajan* makes it distinctly possible that pollen from wild relatives has entered the cultivated gene pool across areas of cultivation in South Asia that overlap with the ranges of closely related wild species such as *C. cajanifolius*. Intra-specific patterns of variation in the wild relatives may be substantial. For traits such as flowering time that varies latitudinal, diverse range-wide collections of wild relatives would be particularly useful for introgressing desirable flowering time variation into cultivated pigeonpea. This could be particularly desirable to adapt it to new regions, or expand the range of seasons in which fresh pigeonpeas are available for markets where the fresh pigeonpeas are in demand.

### Needs for more germplasm collection?

To increase genetic diversity of pigeonpea breeding material, new diversity from wild relatives will be extremely useful. Although we find substantial variation in existing collections, we are certainly under sampling diversity within wild *Cajanus* species. Existing collections are inadequate for in-depth analysis of genetic variation between different *Cajanus* species. In particular, we expect to find substantial variation within species along climatic gradients across India. We advocate for systematic sampling from Madhya Pradesh and Andhra Pradesh to locate the exact geographical location of origin and first domestication event. Sampling from other potential regions would be beneficial to understand the movement of pigeonpea from its origin, and patterns of ongoing hybridization with wild relatives. This would also be helpful in assessing the outcrossing limits of pigeonpea, and allow a determining of isolation distances required for pigeonpea hybrid seed production.

## Supporting Information

Figure S1 **Estimated genome wide (CcLG01 to CcLG11) gene diversity using 875 mapped loci.** “X” axis represents the length of each linkage group (CcLG) in cM and “Y” axis represents the value of gene diversity.
(PDF)

Figure S2 **Population analysis of gene pools of** ***Cajanus*** *a)* Structure results across gene pools. Groups 1, 2, and 3 represent the primary, secondary, and tertiary gene pools *b)* Principal coordinates analysis of domesticated pigeonpea and wild relatives. Red diamonds, primary gene pool; green squares, secondary gene pool; dark blue triangles, tertiary gene pool.
(PNG)

Figure S3 **Analysis of molecular variance (AMOVA) at the continent scale.**
(PNG)

Table S1 **Details on 184** ***Cajanus*** **accessions used for diversity and population analysis.**
(XLSX)

Table S2 **Genotyping data generated using 1,616 PKAM on 184** ***Cajanus*** **accessions.**
(XLS)

Table S3 **Diversity in breeding lines, landraces and wild relatives.**
(XLSX)

Table S4 **Diversity in landraces and wild relatives at the continent scale.**
(XLSX)

Table S5 **Diversity in landraces and wild relatives at the country scale.**
(XLSX)

Table S6 **Diversity in landraces and wild relatives at the province scale with in India.**
(XLSX)
