# Expression of Concern: Genetic Diversity and Demographic History of *Cajanus spp.* Illustrated from Genome-Wide SNPs

**DOI:** 10.1371/journal.pone.0350355

**Published:** 2026-06-01

**Authors:** The PLOS One Editors

The *PLOS One* Editors issue this Expression of Concern because this article [1] is one of a series of articles for which there are concerns about competing interests and the reliability of peer review. The competing interests were not disclosed as required by PLOS policy.

PLOS regrets that the peer review concerns were not identified and addressed before the article was published.

In following up on this matter, PLOS did not evaluate the article’s integrity or scientific validity.
