# Supplementary material for: Genetic Diversity and Demographic History of Cajanus spp. Illustrated from Genome-Wide SNPs
Source: PLoS One. 2014 Feb 12;9(2):e88568. doi: 10.1371/journal.pone.0088568 (PMC3922937; doi:10.1371/journal.pone.0088568)
Supplement: Figure S1 — Estimated genome wide (CcLG01 to CcLG11) gene diversity using 875 mapped loci. “X” axis represents the length of each linkage group (CcLG) in cM and “Y” axis represents the value of gene diversity. (PDF) [file pone.0088568.s001.pdf]

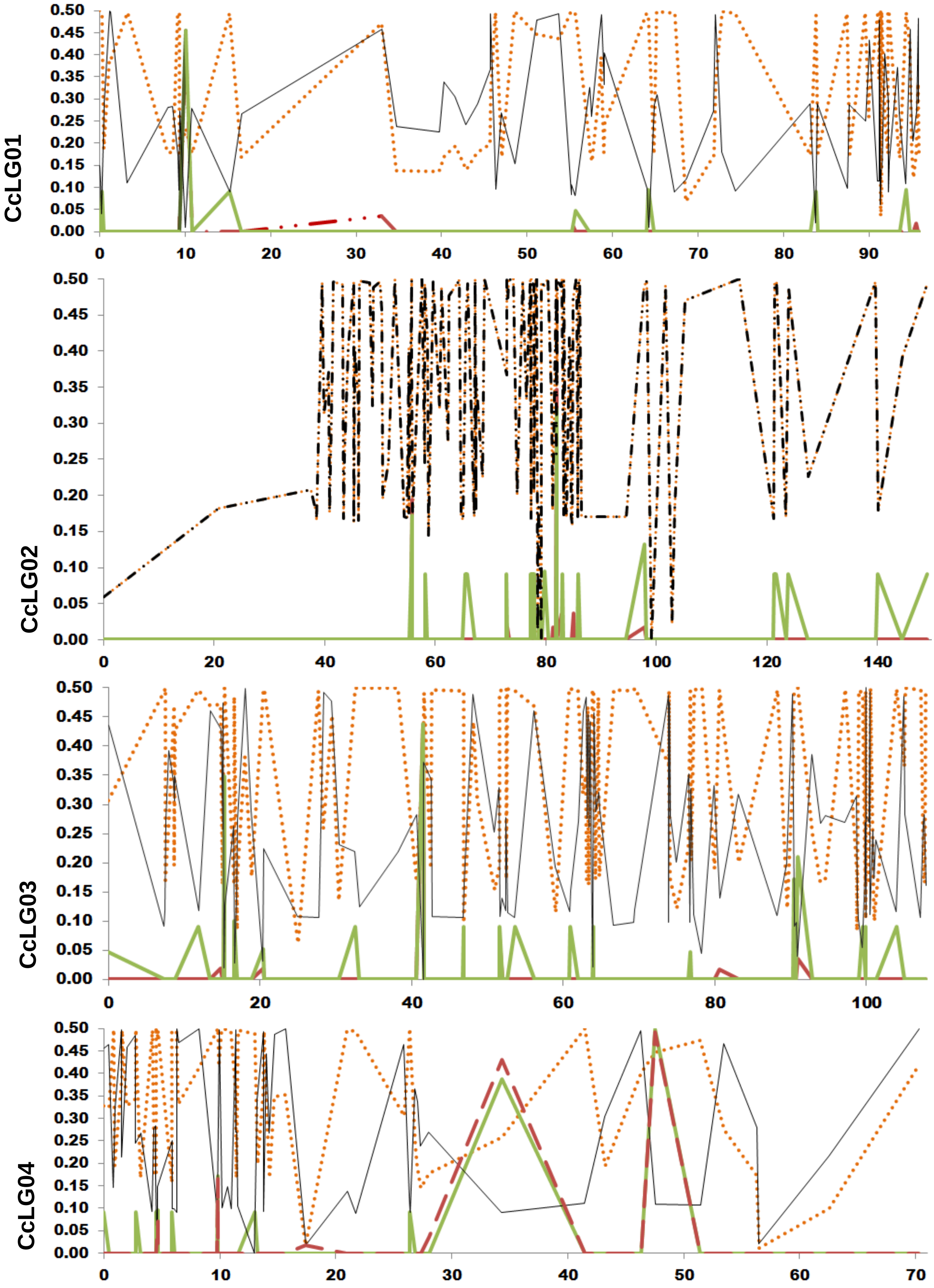

Cont…..

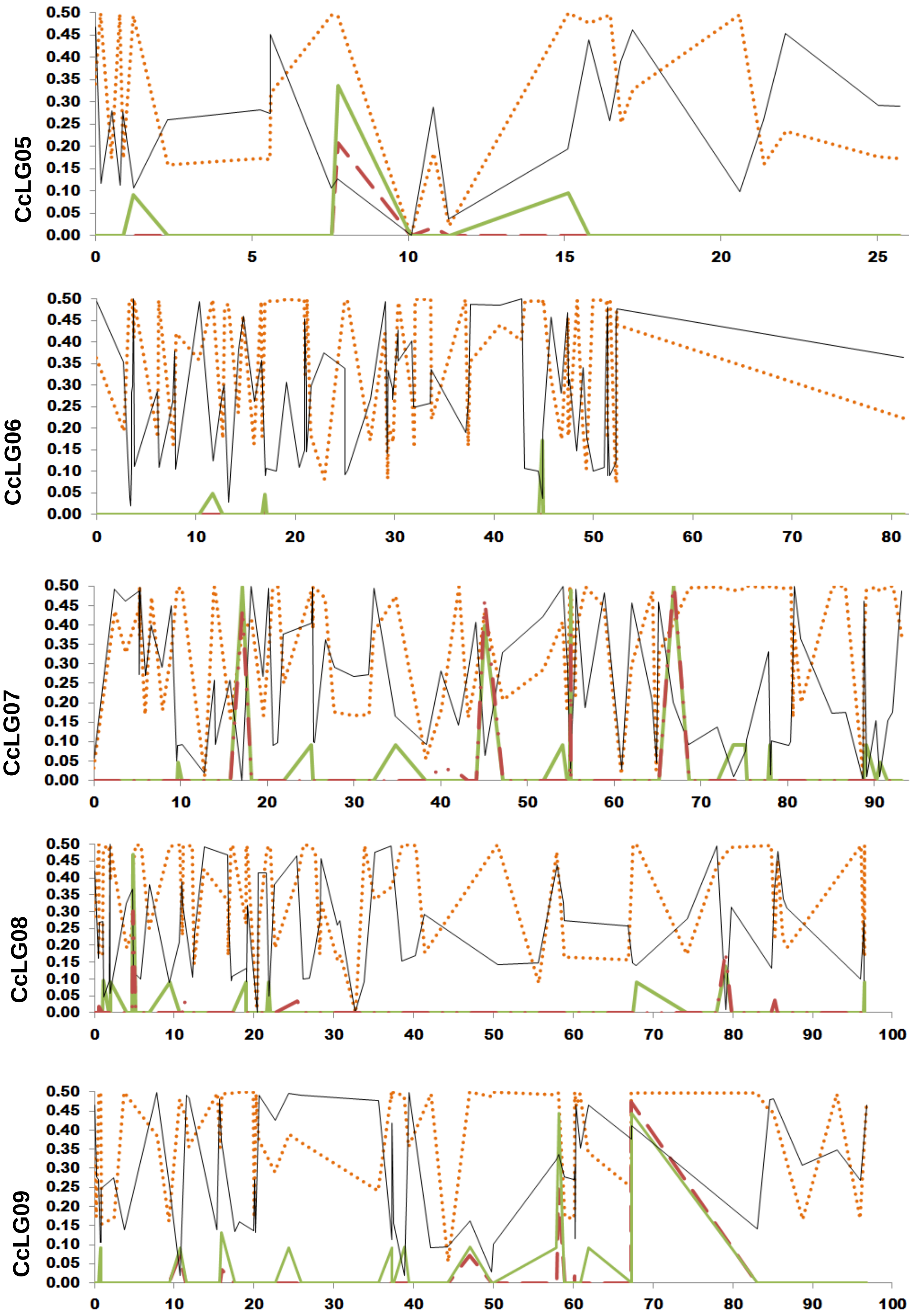
CcLG05
0.50
0.45
0.40
0.35
0.30
0.25
0.20
0.15
0.10
0.05
0.00
0
5
10
15
20
25
CcLG06
0
10
20
30
40
50
60
70
80
CcLG07
0
10
20
30
40
50
60
70
80
90
CcLG08
0
10
20
30
40
50
60
70
80
90
100
CcLG09
0
10
20
30
40
50
60
70
80
90
100

Cont…..

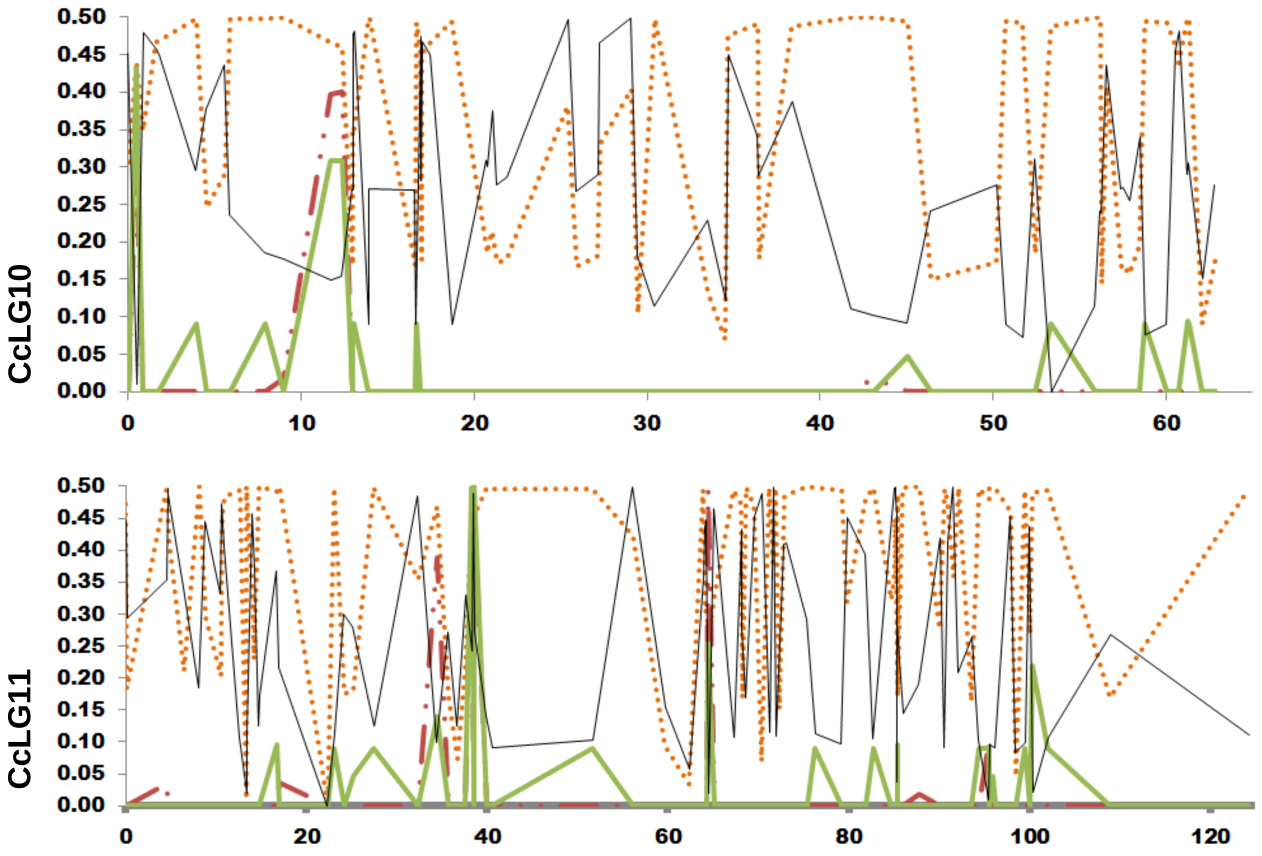

Figure S1: Estimated genome wide (CcLG01 to CcLG11) gene diversity using 875 mapped loci. “X” axis represents the length of each linkage group (CcLG) in cM and “Y” axis represents the value of gene diversity.

Gene diversity across 184 *Cajanus* accessions

Gene diversity across 56 breeding lines

Gene diversity across 21 landraces

Gene diversity across 107 wilds
