# Supplementary figures and images for: Genetic Diversity and Demographic History of Cajanus spp. Illustrated from Genome-Wide SNPs
Source: PLoS One. 2014 Feb 12;9(2):e88568. doi: 10.1371/journal.pone.0088568 (PMC3922937; doi:10.1371/journal.pone.0088568)

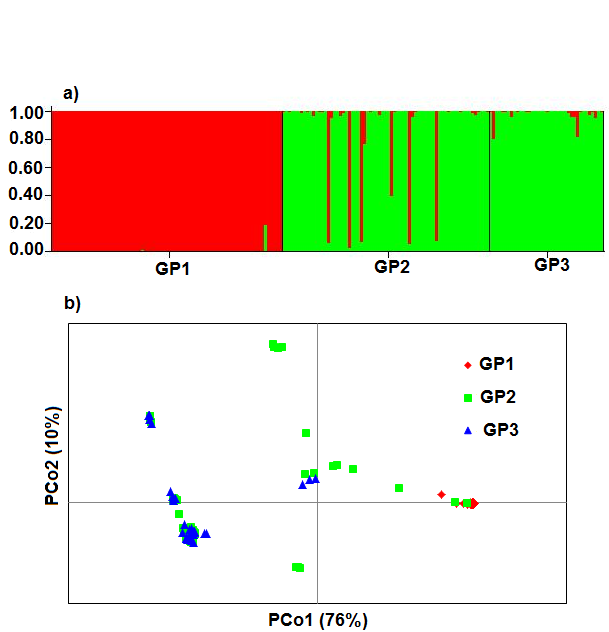

Supplement: Figure S2 — Population analysis of gene pools of Cajanus a) Structure results across gene pools. Groups 1, 2, and 3 represent the primary, secondary, and tertiary gene pools b) Principal coordinates analysis of domesticated pigeonpea and wild relatives. Red diamonds, primary gene pool; green squares, secondary gene pool; dark blue triangles, tertiary gene pool. (PNG) [file pone.0088568.s002.png]

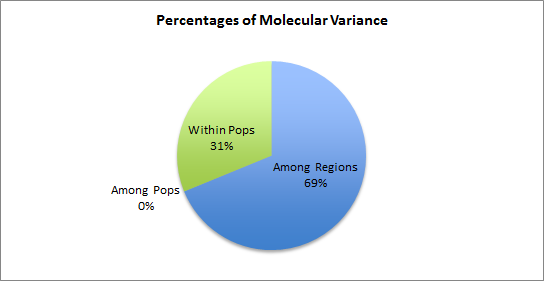

Supplement: Figure S3 — Analysis of molecular variance (AMOVA) at the continent scale. (PNG) [file pone.0088568.s003.png]
